# SFXN1 expression analysis in oral squamous cell carcinoma and its association with the PI3K-AKT-mTOR pathway and immune cell infiltration

**DOI:** 10.1016/j.jobcr.2025.11.010

**Published:** 2025-11-25

**Authors:** Prabhu Manickam Natarajan, Manoj Kumar Karuppan Perumal, Sudhir Rama Varma, Sam Thomas, Ruba Odeh, Remya Rajan Renuka

**Affiliations:** a Department of Clinical Sciences, College of Dentistry, Centre of Medical and Bio-Allied Health Sciences and Research, Ajman University, Ajman, United Arab Emirates; b Centre for Stem Cell Mediated Advanced Research Therapeutics, Saveetha Dental College and Hospitals, Saveetha Institute of Medical and Technical Sciences, Saveetha University, Chennai, Tamil Nadu, 600 077, India

**Keywords:** SFXN1, OSCC, PI3K-AKT-mTOR, Immune infiltration, Biomarker, Bioinformatics

## Abstract

**Background:**

Oral squamous cell carcinoma (OSCC) is a predominant malignancy characterized by aggressive progression and poor prognosis. This study investigated the role of SFXN1 (Sideroflexin 1), a mitochondrial serine transporter, in OSCC using integrated bioinformatic and experimental approaches.

**Objective:**

To analyze the expression, clinical relevance, and functional associations of SFXN1 in OSCC through comprehensive bioinformatic and in vitro investigations.

**Methods:**

Transcriptomic data from TCGA pan-cancer cohorts were analyzed to evaluate SFXN1 expression patterns. The expression levels in OSCC were validated using KB OC cell lines, with clinical correlations assessed for tumor grade, nodal status, and patient survival. Immune infiltration associations and protein‒protein interaction networks were constructed, followed by pathway enrichment analyses. Experimental validation was performed via in vitro assays.

**Results:**

The results revealed that SFXN1 was significantly overexpressed in head and neck squamous cell carcinoma and markedly upregulated in KB cells compared with controls. SFXN1 expression was associated with tumor grade and nodal metastasis, although no significant stage-specific differences were observed. Survival analysis revealed no statistically significant association with overall survival. Immune infiltration analysis indicated modest but significant correlations between SFXN1 and immune cell populations, particularly CD4^+^ T cells. Protein network analysis identified hub genes, including AKT1, BCL2, MTOR, and CASP3. Pathway enrichment implicated SFXN1 is involved in the PI3K-AKT-mTOR and p53 signaling pathways.

**Conclusion:**

These findings highlight the involvement of SFXN1 in cancer-related pathways and its potential role in OSCC, suggesting potential therapeutic targeting opportunities that need further investigation.

## 1 Introduction

Oral cancer (OC) is a widespread malignancy worldwide, with oral squamous cell carcinoma (OSCC) accounting for more than 90 % of all OC cases.^1^, ^2^, ^3^, ^4^ It is particularly prevalent in South Asia and Southeast Asia because of the high consumption of tobacco, areca nuts, and alcohol.^5^, ^6^, ^7^ The 5-year survival rate of OSCC patients is still incredibly low despite advancements in diagnostic and treatment techniques. This is frequently because of late-stage diagnosis, locoregional invasion, and frequent recurrence. This highlights the urgent need to identify reliable diagnostic, prognostic, and therapeutic biomarkers that can be utilized for early detection and treatment.^8^ The molecular alterations that drive OSCC involve the dysregulation of several oncogenes and tumor suppressors, including TP53, EGFR, CDKN2A, and genes regulating the PI3K/AKT/mTOR signaling axis. However, despite extensive studies, current biomarkers have limited predictive power and often lack specificity. In this context, recent studies have focused on mitochondrial metabolism and transport as potential contributors to cancer progression. Mitochondria not only regulate cellular energetics and apoptosis but also actively modulate signaling cascades and redox homeostasis—processes hijacked in tumorigenesis.^9^, ^10^, ^11^

Sideroflexin 1 (SFXN1), a mitochondrial inner membrane protein, belongs to the sideroflexin family and functions primarily as a serine transporter crucial for one-carbon metabolism.^12^^,^^13^ This pathway is central to nucleotide synthesis, methylation, and redox balance—metabolic needs that are often amplified in cancer cells. In addition to its metabolic role, SFXN1 has been linked to oncogenesis in emerging studies, although its role remains poorly characterized. Numerous cancers, including colorectal, liver, and breast cancers, exhibit abnormal expression of SFXN1, indicating that SFXN1 has a shared carcinogenic function across tumor types. Given the importance of metabolic reprogramming in cancer progression and the emerging involvement of SFXN1 in malignancies, we hypothesized that SFXN1 might play a crucial role in OSCC.^14^ This study explored the expression profile, clinical correlations, and functional significance of SFXN1 in OSCC through integrated bioinformatic analysis and experimental validation, with a particular focus on its associations with key signaling pathways and immune cell infiltration. We utilized data from the TCGA database to assess SFXN1 expression across cancers, specifically in HNSC, which is closely related to OSCC. Correlation analyses were performed to determine relationships with clinical parameters such as tumor stage, nodal metastasis, and histologic grade.^15^ Additionally, immune infiltration and survival associations were evaluated to assess their prognostic value. Furthermore, we explored the SFXN1 molecular interaction landscape via STRING and Cytoscape to identify coregulatory partners and construct protein‒protein interaction networks. Coexpression analyses of key apoptotic and signaling genes were performed to decipher the mechanistic pathways potentially influenced by SFXN1. To corroborate our bioinformatic findings, we performed in vitro validations in KB OC cells. SFXN1 expression was quantified at both the mRNA and protein levels through RT‒PCR. Immunofluorescence staining confirmed the intracellular localization of this protein, strengthening the hypothesis of its mitochondrial involvement. These combined analyses provide compelling evidence for the diagnostic and potential therapeutic importance of SFXN1 in OSCC.

## 2 Materials and methods

### 2.1 SFXN1 expression status from TCGA datasets

The expression profiles of SFXN1 across multiple cancer types were analyzed using the TIMER 2.0 platform, and the transcript levels were quantified in TPM units. For OC analysis, SFXN1 mRNA data and clinical variables, including metastatic status and tumor stage, were retrieved from UALCAN using TCGA datasets comprising 520 tumor specimens and 44 adjacent normal controls to assess differential expression patterns. Survival data stratified by OC stage were obtained through Kaplan–Meier (KM) plotter analysis. Protein-level visualization was enhanced using immunohistochemical data from the Human Protein Atlas (https://www.proteinatlas.org/).

### 2.2 SFXN1 co-networking

Protein interaction networks were reconstructed utilizing the STRING database (https://string-db.org/) to identify SFXN1-associated molecular partners. Network topology was visualized and analyzed through Cytoscape software (v3.5.1), where interaction confidence was represented by edge weighting based on cumulative scoring metrics.^16^ Hub node identification and network clustering were performed using the CytoHubba extension to delineate functional modules. The resulting network architecture revealed interconnected gene clusters corresponding to separate molecular networks and regulatory circuits implicated in OC pathogenesis.

### 2.3 SFXN1 tumor immune infiltration and survival analysis

Immune microenvironment characterization was performed TIMER 2.0 to evaluate SFXN1-correlated infiltration patterns in oral malignancies. Three primary lymphocyte populations (B cells, CD4^+^ T cells, and CD8^+^ T cells) were quantified based on transcriptomic signatures within TIMER datasets. Comparative analysis between the SFXN1-high and SFXN1-low patient cohorts was conducted through Kaplan‒Meier survival curves, box plot distributions, and correlation matrices. Prognostic significance was determined by calculating cumulative survival percentages across temporal intervals, emphasizing the interplay between SFXN1 expression and immune cell dynamics in patient outcomes. Co-expression relationships were established using Spearman correlation coefficients (R) derived from the TIMER computational framework.

### 2.4 Functional annotations

Comprehensive functional and pathway enrichment analyses were performed to reveal the biological relevance of SFXN1 and its associated gene clusters. The Enrichr database was utilized for systematic and consistent gene annotation to identify key biological functions and pathways that were significantly enriched in relation to upregulated biomolecules. The analysis categorized genes into distinct Gene Ontology (GO) terms, including molecular function (MF), cellular component (CC), and biological process (BP) terms. Additionally, KEGG pathway analyses were conducted to identify critical signaling and metabolic pathways significantly enriched in association with identified gene targets in OC.

### 2.5 Cell culture

KB human cells were acquired from NCCS, India, and grown under standard conditions in a CO_2_ incubator. The cells were maintained in Dulbecco's modified Eagle's medium (DMEM) supplemented with 10 % fetal bovine serum (FBS) and 1 % penicillin‒streptomycin to ensure healthy growth. SFXN1 was subsequently transfected into KB cells, and successful integration was confirmed through GFP immunofluorescence.

#### 2.5.1 Cell viability assay

The viability of KB OC cells was assessed via the MTT dye. Approximately 3000 cells were seeded into each well of a 96-well plate.^17^ Following treatment with either WT (control) or SFXN1 KO, MTT solution (5 mg/mL) was added to the wells for time-dependent analysis, and the mixture was incubated for 4 h. After incubation, the resulting formazan crystals were dissolved in 100 μL of DMSO per well. The optical density (OD) was measured at 570 nm using a Bio-Rad microplate reader. Additionally, morphological changes in the cells were observed under an Olympus inverted microscope equipped with a Zeiss camera.

#### 2.5.2 Flow cytometry analysis

KB cells transfected with either WT (control) or SFXN1-KO cells were seeded into 6-well plates at a density of 2 × 10^5^ cells per well and incubated for 24 h.^18^ For cell cycle analysis, cells were stained with propidium iodide (PI) and incubated at room temperature for 10 min prior to analysis. For apoptosis detection, annexin V-FITC/PI dual staining was utilized. DNA content and apoptosis were assessed using flow cytometry on a BD FACS Calibur system, and the resulting data were analyzed using Cell Quest Pro software (version 3.2.1, Becton Dickinson, USA).

#### 2.5.3 Immunofluorescence staining

KB cells with SFXN1 knockout (5 × 10^4^ cells/well) were cultured in 12-well plates, washed with PBS, and fixed with 4 % paraformaldehyde for morphological preservation. Cell viability assessment was conducted through acridine orange/ethidium bromide dual fluorescent labelling to distinguish viable from apoptotic cells via nuclear characteristics. Stained cells were suspended in 1 mL chilled PBS and examined at 20 × magnification using Olympus fluorescence microscopy (Tokyo, Japan) with an integrated Zeiss imaging system.

### 2.6 Gene expression by RT-PCR

KB cells (2 × 10^6^ cells/well) were cultured overnight in 6-well plates. RNA isolation was performed using TRIR extraction reagent (Abgene, UK), and the RNA was quantified via spectrophotometry.^19^ cDNA synthesis was conducted from 2 μg of total RNA following established protocols. For quantitative PCR amplification, Takara SYBR Green chemistry with gene-specific oligonucleotides was used. The amplification parameters included initial denaturation at 95 °C (5 min), followed by 30 cycles of 95 °C (5 s) and 56–65 °C (8 s). Oligonucleotide sequences: SFXN1 - forward: 5′-ACCAGTCCTTCAATGCCGTCGT-3′, reverse: 5′-GAGTCCTAGAGCTGTTGCTACG-3'; β-actin - forward: 5′-AGAGCTACGAGCTGCCTGAC-3′, reverse: 5′-AGCACTGTGTTGGCGTACAG-3'.

### 2.7 Statistical analysis

Data analysis was performed using R programming language. Log-rank tests and p-value calculations were conducted via GraphPad Prism v8. Kaplan‒Meier survival curves were generated for survival analysis. Statistical significance: ∗p < 0.05, ∗∗p < 0.001, ∗∗∗p < 0.0001.

## 3 Results

### 3.1 Clinical relevance of SFXN1 expression in head and neck squamous cell carcinoma (HNSC)

**Fig. 1:**
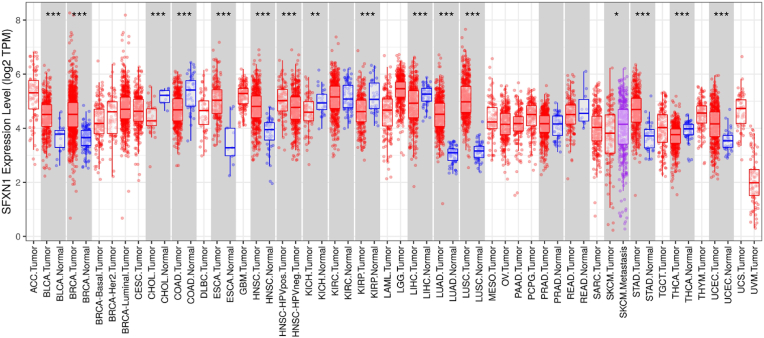
The pan-cancer expression boxplot shows significantly elevated SFXN1 expression in tumor samples compared to normal tissues across multiple cancer types. Notably, in HNSC (highlighted in grey), SFXN1 expression is significantly higher in tumor tissue than in normal counterparts (p < 0.001), suggesting a potential oncogenic role in OC.

**Fig. 2:**
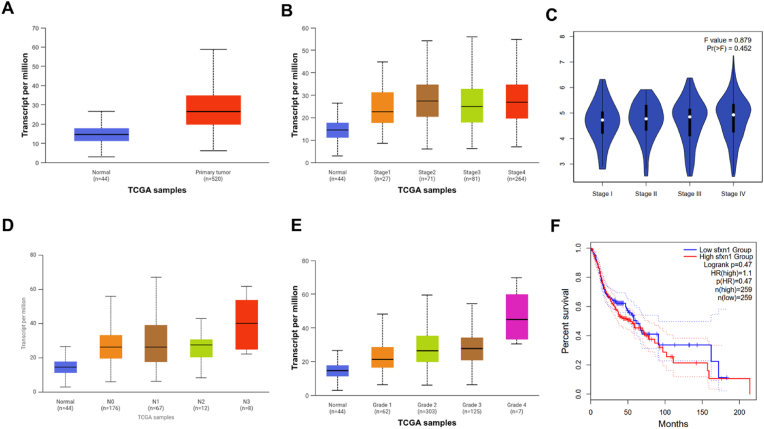
**Expressional status of SFXN1 in HNSC-TCGA datasets**. (A) Boxplots reveal higher SFXN1 expression in primary tumor samples versus normal tissues in OSCC. (B & C) Stage-wise and nodal (N) staging analysis showed a progressive increase in expression with higher stages and nodal involvement. (D) ANOVA (violin plot) across clinical stages did not show statistical significance (p > 0.05). (E) Grade analysis showed a steady increase in SFXN1 expression from Grade 1 to Grade 4, aligning with tumor aggressiveness. (F) Kaplan-Meier survival plots indicated a trend of poorer prognosis in patients with elevated SFXN1 expression (HR = 1.1), though not statistically significant.

The data presented in Fig. 1 elucidate the clinical implications of SFXN1 expression in carcinoma (HNSC) on the basis of TCGA datasets. In the first panel, significantly greater SFXN1 transcript levels were detected in primary tumor tissues (n = 520) than in adjacent normal tissues (n = 44), indicating that SFXN1 is markedly overexpressed in HNSC tumors, as shown in Fig. 2A. Stratification by cancer stage revealed no statistically significant differences in SFXN1 expression across stages I-IV (F = 0.879, p = 0.452), indicating that SFXN1 overexpression occurs regardless of tumor stage rather than correlating with disease progression, as shown in Fig. 2B and C. However, nodal status analysis revealed a clear upward trend in SFXN1 levels with increasing lymph node involvement (N0--N3), with N3 showing the highest expression, implying that SFXN1 could be associated with nodal metastasis (Fig. 2D). The correlation with tumor grade further strengthened this association, as patients with higher histological grades (grades 3 and 4) presented significantly greater SFXN1 expression than did those with lower grades and normal tissues, supporting its role in aggressive tumor phenotypes (Fig. 2E). Survival analysis via Kaplan‒Meier plots revealed a modest but noticeable reduction in overall survival in patients with high SFXN1 expression (HR = 1.1; p = 0.47), although this difference was not statistically significant, as depicted in Fig. 2F. Nonetheless, this trend suggests a potential association between high SFXN1 levels and poor prognosis.

### 3.2 Network analysis of SFXN1 in HNSC

**Fig. 3:**
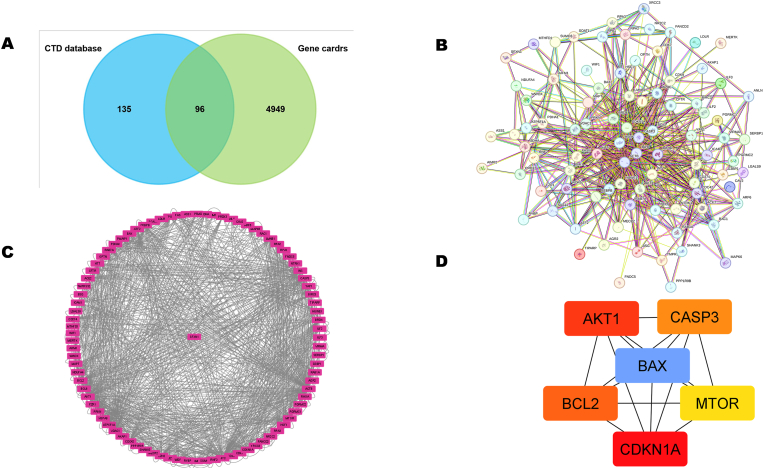
**SFXN1's networking association**. (A) The Venn diagram reveals 96 overlapping genes related to SFXN1 from the CTD and Gene Cards databases. (B) The STRING-based PPI network and (C) Cytoscape interaction plot show dense connectivity, indicating SFXN1's involvement in vital cellular pathways. (D) Key hub genes include AKT1, BCL2, CASP3, MTOR, CDKN1A, and BAX, central to apoptosis and cell survival regulation.

Integrative network analysis of SFXN1 in head and HNSC highlights its involvement in key oncogenic and apoptotic signaling pathways. Venn diagram analysis revealed 96 overlapping genes between the Comparative Toxicogenomics Database (CTD) and the GeneCards database, representing a shared pool of SFXN1-associated genes, as shown in Fig. 3A. Protein‒protein interaction (PPI) networks constructed via STRING and visualized via Cytoscape illustrate a densely interconnected network, suggesting robust functional associations between SFXN1 and several core regulatory proteins, as depicted in Fig. 3B. The central nodes identified in the PPI analysis included critical oncogenes and apoptosis regulators such as AKT1, BCL2, MTOR, CASP3, CDKN1A, and BAX. These hub genes are key players in cancer-relevant pathways, particularly the PI3K-AKT-mTOR and p53 signaling cascades (Fig. 3C). SFXN1 appears to be functionally linked with cell survival and growth promotion genes (AKT1, BCL2, MTOR) and pro-apoptotic components (BAX, CASP3), indicating its dual role in maintaining mitochondrial homeostasis and regulating cell fate decisions in tumor cells (Fig. 3D). The circular Cytoscape network map, which places SFXN1 at the core, reflects its high connectivity and potential regulatory influence on numerous cancer-associated genes.

### 3.3 SFXN1 immune infiltration and survival in HNSC

**Fig. 4:**
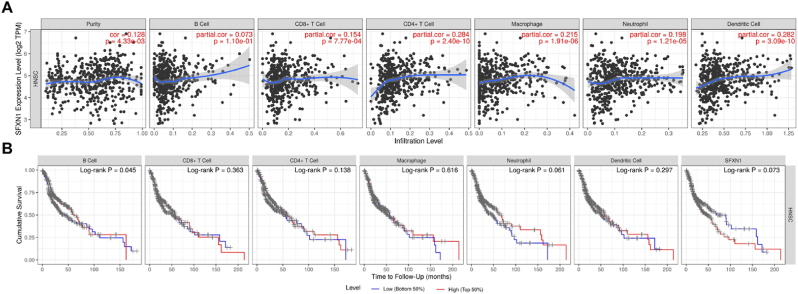
**SFXN1 associated with immune cells**. (A) Correlation and (B) survival analyses with immune infiltration highlight a significant association between SFXN1 and immune cell levels. CD4^+^ T cells, dendritic cells, and macrophages show positive correlation (p < 0.001), and survival analysis stratified by immune infiltration levels suggests potential immunomodulatory roles of SFXN1, particularly with B cells (Log-rank p = 0.045).

The data presented highlight the relationship between SFXN1 expression levels and immune cell infiltration in HNSC, along with its impact on survival outcomes. The analysis revealed significant associations between SFXN1 expression and various immune cell types, including CD4^+^ T cells, macrophages, and dendritic cells, as indicated by partial correlation coefficients (partial CVs) and p values. Notably, CD4^+^ T cells were most strongly correlated with SFXN1 expression (p < 0.284, p = 2.40e-10), but these correlation coefficients represent modest associations that require mechanistic validation. Macrophages and dendritic cells also exhibited significant correlations (partially cov = 0.215 and 0.188, respectively), further implicating SFXN1 in immune regulation (Fig. 4A). Survival analysis, however, presents mixed results. While B cells were marginally significantly associated with survival (log-rank P = 0.045), other immune cell types, such as CGA-T cells and macrophages, were not significantly correlated with survival (log-rank P = 0.353 and 0.616, respectively). Intriguingly, SFXN1 itself tended to significantly affect survival outcomes (log-rank P = 0.073) (Fig. 4B).

### 3.4 SFXN1 Gene Ontology and pathway prediction

**Fig. 5:**
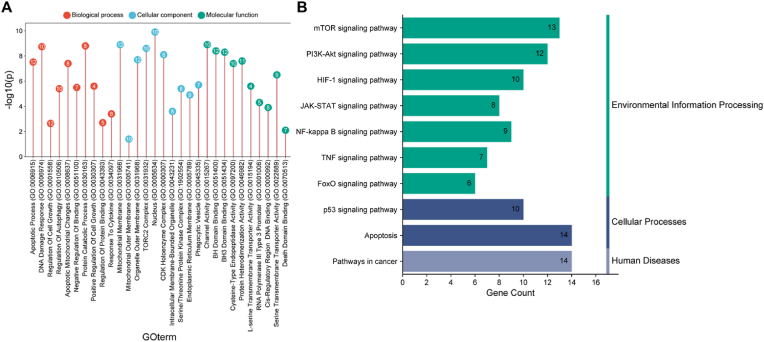
**Functional annotations**. (A) GO and (B)KEGG pathway enrichment plots reveal that SFXN1-associated genes are enriched in apoptotic processes, mitochondrial membrane components, and serine/threonine kinase activities. KEGG analysis shows involvement in the PI3K-AKT, mTOR, JAK-STAT, p53, and FoxO signaling pathways, indicating a central role in cancer-related signaling.

**Fig. 6:**
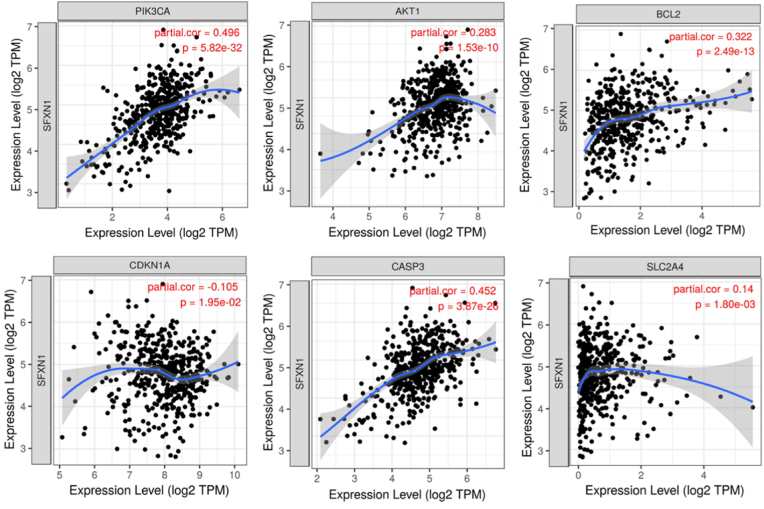
Scatterplots confirm strong positive correlations between SFXN1 and PIK3CA (r = 0.496), CASP3 (r = 0.452), BCL2, and AKT1. These suggest co-expression and potential regulatory interaction with apoptosis and survival-related genes. A negative correlation was observed with CDKN1A (r = −0.105), reinforcing its oncogenic role.

GO pathway analyses revealed the multifaceted role of SFXN1 in critical biological processes, cellular components, and molecular functions. SFXN1 is involved in the DNA damage response, the regulation of cell growth, and responses to cytokines, suggesting its involvement in maintaining genomic stability and modulating immune and cellular proliferation pathways. Notably, SFXN1 is associated with mitochondrial mechanisms, and intracellular metric cells, suggesting its potential role in cellular metabolism and structural organization. The presence of SFXN1 in the nucleus, endoplasmic reticulum membrane, and serine/threonine protein kinase complex further underscores its regulatory functions in signaling and protein interactions, as shown in Fig. 5A and Table 1. Pathway analysis highlighted the connection of SFXN1 to key signaling cascades, including the mTOR, PI3K-Akt, and HIF-1 pathways, which are pivotal in cancer progression, metabolism, and the hypoxia response. Its association with the JAK-STAT and NF-kappa B pathways suggests a role in immune regulation and inflammation, whereas its links to the p53 pathway and apoptosis indicate potential tumor-suppressive functions. Additionally, the involvement of SFXN1 in the FoxO signaling pathway highlights its influence on cellular stress responses and longevity, as shown in Fig. 5B. Moreover, scatterplot analysis revealed strong positive correlations between SFXN1 and key genes such as PIK3CA (r = 0.496), CASP3 (r = 0.452), BCL2, and AKT1, indicating potential co-expression and regulatory interactions with genes involved in apoptosis and cell survival. Conversely, a negative correlation with CDKN1A (r = −0.105) further supported the oncogenic role of SFXN1, as shown in Fig. 6.

**Table 1:** Gene ontologies.

GO Terms	-log10(p)	count
Apoptotic Process (GO:0006915)	7.52	12
DNA Damage Response (GO:0006974)	8.74	10
Regulation Of Cell Growth (GO:0001558)	2.64	12
Regulation Of Autophagy (GO:0010506)	5.4	10
Apoptotic Mitochondrial Changes (GO:0008637)	7.4	8
Negative Regulation of Binding (GO:0051100)	5.5	7
Protein Catabolic Process (GO:0030163)	8.8	8
Positive Regulation of Cell Growth (GO:0030307)	5.6	4
Regulation Of Protein Binding (GO:0043393)	2.7	5
Response To Cytokine (GO:0034097)	3.4	8
Mitochondrial Membrane (GO:0031966)	8.9	12
Mitochondrial Outer Membrane (GO:0005741)	1.4	10
Organelle Outer Membrane (GO:0031968)	7.7	12
TORC2 Complex (GO:0031932)	8.6	10
Nucleus (GO:0005634)	9.9	10
Cyclin-Dependent Protein Kinase Holoenzyme Complex (GO:0000307)	8.1	8
Intracellular Membrane-Bounded Organelle (GO:0043231)	3.6	8
Serine/Threonine Protein Kinase Complex (GO:1902554)	5.4	8
Endoplasmic Reticulum Membrane (GO:0005789)	4.9	9
Phagocytic Vesicle (GO:0045335)	5.7	7
Channel Activity (GO:0015267)	8.9	10
BH Domain Binding (GO:0051400)	8.4	12
BH3 Domain Binding (GO:0051434)	8.3	12
Cysteine-Type Endopeptidase Activity Involved in Execution Phase of Apoptosis (GO:0097200)	7.4	10
Protein Heterodimerization Activity (GO:0046982)	7.6	11
L-serine Transmembrane Transporter Activity (GO:0015194)	5.6	4
RNA Polymerase III Type 3 Promoter Sequence-Specific DNA Binding (GO:0001006)	4.3	5
RNA Polymerase III Cis-Regulatory Region Sequence-Specific DNA Binding (GO:0000992)	3.9	8
Serine Transmembrane Transporter Activity (GO:0022889)	6.5	9
Death Domain Binding (GO:0070513)	2.1	7

### 3.5 Deletion of SFXN1 reduces cell proliferation and induces cell death in KB cells

**Fig. 7:**
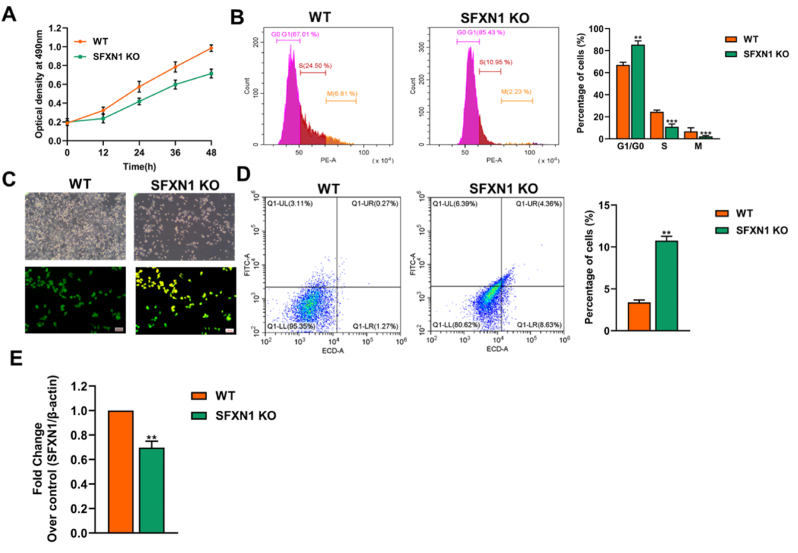
**Functional characterization of SFXN1 knockout (KO) in KB oral squamous carcinoma cells**. (A) Cell proliferation assay using MTT at different time intervals (0–48 h) shows a significant reduction in cell viability in SFXN1 KO cells compared to wild-type (WT) cells. (B) Flow cytometric cell cycle analysis of WT and SFXN1 KO cells using PI staining indicates increased G0/G1 phase arrest and reduced S and M phase populations in KO cells. (C) Phase-contrast microscopy images and AO/EtBr dual staining show increased apoptotic morphology in SFXN1 KO cells (yellow/orange nuclei) compared to WT (green nuclei). (D) Annexin V/PI staining followed by flow cytometry further confirms enhanced apoptosis in SFXN1 KO cells. (E) qPCR analysis shows decreased SFXN1 mRNA expression in KO cells normalized to β-actin.

Experimental analysis revealed the impact of SFXN1 knockout (KO) on the biological behavior of KB OSCC cells. The MTT assay revealed a significant reduction in cell the viability of SFXN1-KO cells compared with WT cells after 48 h, suggesting that SFXN1 plays a role in promoting cellular proliferation, as shown in Fig. 7A. Cell cycle analysis via flow cytometry demonstrates a significant arrest at the G1/G0 phase in SFXN1-deficient cells, with a marked reduction in the S and M phases. These findings indicate that SFXN1 is crucial for progression through the cell cycle and that its depletion leads to cell cycle blockade, as shown in Fig. 7B. Morphological evaluation under phase-contrast microscopy indicates decreased cell density and altered cellular morphology in KB cells, which is consistent with impaired growth (Fig. 7C). Fluorescence imaging with acridine orange/ethidium bromide staining reveals increased apoptotic features in KO cells, as evidenced by prominent nuclear fragmentation and membrane blebbing (Fig. 7C). Furthermore, apoptosis analysis via Annexin V/PI staining shows a substantial increase in early and late apoptotic populations in the KO cells, highlighting the role of SFXN1 in cell survival and resistance to apoptosis, as shown in Fig. 7D. Gene and protein expression analyses confirmed the successful knockout of SFXN1, with both RT‒qPCR as depicted in Fig. 7E. These findings collectively suggest that SFXN1 is functionally involved in promoting cell proliferation, preventing apoptosis, and facilitating cell cycle progression in OC cells.

## 4 Discussion

This comprehensive analysis focused on characterizing SFXN1 expression patterns and their molecular associations in OSCC rather than establishing their prognostic utility. Our findings provide insights into the role of SFXN1 in cancer biology through pathway analysis and functional validation. OC development involves the dysregulation of several critical signaling pathways, including the PI3K/AKT/mTOR, EGFR, MAPK, STAT3, and Wnt/β-catenin pathways, which collectively contribute to cellular transformation, angiogenesis, resistance to apoptosis, and immune evasion.^10^^,^^12^^,^^14^ Aberrations in tumor suppressor genes such as TP53 and the overexpression of oncogenes such as Cyclin D1, MYC, and VEGF further drive carcinogenesis. Epigenetic alterations and noncoding RNAs, including miRNAs and lncRNAs, also play crucial roles in modulating gene expression and tumor behavior. Current treatment strategies for OC typically involve surgical resection, often followed by radiotherapy and chemotherapy using agents such as cisplatin and 5-fluorouracil. However, these conventional approaches are associated with significant side effects and limited efficacy in advanced stages.^20^ Molecular-targeted gene therapy has emerged as a promising alternative, focusing on silencing or correcting specific oncogenes or restoring the function of tumor suppressor genes. Techniques such as siRNA-mediated gene silencing, CRISPR-Cas9 gene editing, and vector-based gene delivery systems are under exploration to selectively target molecular drivers of OC.^21^, ^22^, ^23^ Additionally, computational approaches have revolutionized the landscape of OC research by enabling bioinformatic analysis, drug repurposing, molecular docking, and network pharmacology. These tools facilitate the identification of novel biomarkers, therapeutic targets, and small molecule inhibitors through in silico screening and modeling.^24^ This comprehensive analysis focused on characterizing SFXN1 expression patterns and their molecular associations in OSCC rather than establishing their prognostic utility. Our findings provide insights into the role of SFXN1 in cancer biology through pathway analysis and functional validation, although several methodological limitations must be acknowledged.

The present study underscores the clinical significance of SFXN1 expression HNSC by analyzing transcriptomic data from the TCGA dataset. Compared with that in adjacent normal tissues, SFXN1 was notably overexpressed in primary tumor tissues, indicating its potential involvement in HNSC pathogenesis. SFXN1 has been linked to mitochondrial iron metabolism and tumor growth in other malignancies.^25^ This study expands the investigation of SFXN1 in head and neck cancer, although its specific role in OSCC biology requires further clarification. While SFXN1 expression was associated with tumor grade and nodal involvement, no significant stage-specific differences were observed (F = 0.879, p = 0.452), indicating that SFXN1 overexpression occurs regardless of tumor stage rather than being correlated with disease progression. While survival analysis did not reveal significant associations with patient outcomes (HR = 1.1, p = 0.47), the consistent overexpression of SFXN1 in tumor tissues and its associations with certain tumor characteristics suggest biological functions that merit further investigation.^26^

The networking analysis revealed that SFXN1 is highly associated with key oncogenic and apoptotic regulators, such as AKT1, MTOR, BCL2, CASP3, and BAX, in HNSC. SFXN1 is positioned centrally in the PPI network and plays a dual role in tumor progression. Additionally, SFXN1 is functionally connected with key oncogenic regulators, positioning it as a central node in cancer-related molecular networks rather than a prognostic indicator. SFXN1 has been implicated in mitochondrial iron metabolism and immune response modulation in other cancer types, such as lung cancer, but this study expands its importance to immune infiltration in HNSC, particularly highlighting its link with CD4^+^ T cells and possible impact on clinical outcomes.^27^

This study provides valuable insights into the immunological role of SFXN1 in HNSC by analyzing its correlation with immune cell infiltration and survival outcomes. The data indicate that SFXN1 expression is significantly associated with the presence of CD4^+^ T cells, macrophages, and dendritic cells, with CD4^+^ T cells showing a positive correlation (partial cov = 0.284, p = 2.40e−10). These results suggest a potential immunomodulatory function of SFXN1 within the tumor microenvironment, possibly by influencing immune cell recruitment or activation. Although survival analysis revealed only a marginal association between B-cell infiltration and overall survival (p = 0.045), SFXN1 itself tended toward prognostic relevance (p = 0.073). Moreover, GO and pathway analyses revealed that SFXN1 is a multifunctional gene involved in the DNA damage response, cytokine signaling, and cell growth regulation. It localizes to the nucleus, ER membrane, and kinase complexes, suggesting roles in signaling and structural organization. SFXN1 is linked to cancer-related pathways, including the mTOR, PI3K-Akt, HIF-1, JAK-STAT, NF-κB, and p53 pathways, highlighting its role in tumor progression, immune regulation, apoptosis, and metabolism.^28^

Previous reports in other cancer types, notably ovarian cancer, have consistently demonstrated that SFXN1 plays a pivotal role in supporting tumor growth, metabolic regulation, and mitochondrial function. This study revealed that SFXN1 facilitates the transport of serine into mitochondria, which in turn supports one-carbon metabolism, a critical pathway for nucleotide biosynthesis, methylation reactions, and redox balance.^29^ This metabolic support enables cancer cells to sustain rapid proliferation and resist oxidative stress. Similarly, in breast cancer, the study demonstrated that the overexpression of SFXN1 is correlated with poor prognosis and aggressive tumor behavior.^30^ Mechanistically, SFXN1 was found to influence the tumor immune microenvironment by modulating the infiltration of immune cells, including T cells and macrophages, and activating inflammatory and oncogenic pathways.^31^ This study demonstrated the functional effects of SFXN1 manipulation in KB cells, including impacts on cell viability, cell cycle progression, and apoptosis, although the interpretation of these findings is limited by the cell line used.

This study has notable limitations, particularly our exclusive use of KB cells for experimental validation. While KB cells have been identified as HeLa derivatives rather than authentic oral carcinoma cells, they remain widely used in OC research.^32^ Despite their continued use in the field, the HeLa origin of KB cells introduces uncertainty regarding the direct applicability of our findings to OSCC biology. The observed cellular effects may reflect characteristics of the HeLa lineage rather than authentic OSCC responses, suggesting that our results should be interpreted as preliminary functional insights that would benefit from validation in authenticated OSCC cell lines. Additionally, survival analysis did not demonstrate statistically significant associations with patient outcomes (HR = 1.1, p = 0.47), indicating that SFXN1 expression levels do not predict survival in this dataset. The immune infiltration analysis revealed statistically significant but modest correlations with various immune cell populations (correlation coefficients ranging from 0.188 to 0.284). While these correlations suggest potential associations between SFXN1 and immune cell infiltration patterns, the correlational nature of these findings means that they represent associations rather than established functional relationships.^33^ The absence of stage-specific expression differences indicates that SFXN1 overexpression occurs independently of tumor stage progression. These findings collectively suggest that while SFXN1 is consistently overexpressed in OSCC and participates in relevant molecular networks, further mechanistic studies using authenticated cell line models and larger patient cohorts would help clarify its specific role in OSCC biology and potential clinical applications.

## 5 Conclusion

This study provides substantial evidence supporting SFXN1 as a promising biomarker in oral squamous cell carcinoma (OSCC). Bioinformatic analyses demonstrated that SFXN1 is significantly overexpressed in OSCC and is positively correlated with advanced clinical features such as tumor grade and nodal metastasis. Immune cell correlation analyses revealed that SFXN1 may influence by the tumor immune microenvironment. Furthermore, functional enrichment and co-expression with critical regulators such as AKT1, BCL2, and CASP3 suggest its involvement in key cancer signaling pathways such as the PI3K-AKT, mTOR, and p53 pathways. Preliminary experimental validations in KB cells suggest functional effects, although validation in authenticated OSCC cell lines is needed to confirm OSCC-specific relevance. Strategies targeting SFXN1 could offer novel avenues for therapeutic intervention, warranting further investigation in clinical and translational cancer research.

## Author contributions

Prabhu Manickam Natarajan contributed to conceptualization, supervision, project administration, and funding acquisition. Manoj Kumar Karuppan Perumal was involved in methodology, investigation, data curation, and writing of the original draft. Sudhir Rama Varma contributed to formal analysis, validation, and resources. Sam Thomas handled software, data visualization, and writing – review and editing. Ruba Odeh contributed to literature review, data interpretation, and writing – review and editing. Remya Rajan Renuka contributed to conceptualization, methodology, supervision, and writing – review and editing.

## Ethics statement

This article does not contain any studies with human participants or animals performed by any of the authors.

## Sponsorship

This research did not receive any specific grant from funding agencies in the public, commercial, or not-for-profit sector's.

## Ethical clearance

The authors confirm that ethical clearance was not applicable for this study.

## Funding

The authors declare that no funds, grants, or other support were received during the preparation of this manuscript.

## Declaration of competing interest

The authors declare that they have no known competing financial interests or personal relationships that could have appeared to influence the work reported in this paper.
